# The College of Nursing, Limited

**Published:** 1916-04-29

**Authors:** Arthur Stanley

**Affiliations:** Chairman of the Council.


### THE COLLEGE OF NURSING, LIMITED.

**By the Honourable ARTHUR STANLEY, M.P., M.V.O., Chairman of the Council.**

A letter, dated April 18, 1916, addressed to the Hospitals, Infirmaries, and Nurse-Training Schools throughout the Country.

The response to the letter which I addressed on the 1st instant to many of the hospitals, infirmaries, and nurse-training schools throughout the country was so prompt and widespread that at the Conference held a week later at St. Thomas's Hospital more than two hundred representatives were present from England and Wales, Scotland and Ireland. I then took the opportunity of explaining the circumstances which led to the establishment of the College of Nursing, and I laid stress upon the desire of the Council to form as speedily as possible such a Consultative Board as would be representative of all parts of the United Kingdom and of all branches of nursing.

### Representatives on the Consultative Board.

The Council has learnt with regret that, as through unavoidable reasons the notice I was able to give of the Conference was somewhat short and some of the letters miscarried, many nursing authorities failed to receive my communication in time to take formal action upon it. I am, therefore, desired to give expression to the earnest hope of the Council that your Board of Management may be pleased within the next month to nominate not more than two representatives upon the Consultative Board of the College, and, as some guide to the lines on which we are working, I am amplifying certain points in the constitution of the College to which allusion only was made in my letter of December 30, 1915.

### The Registration of the College.

To the reasons which induced me to lay before the managers of hospitals and infirmaries proposals for the establishment of a College of Nursing at the time I did I need not recur; but I ought perhaps to say that, whilst it was originally my intention to ask the Board of Trade to register the College without the word "Limited," I found on inquiry that such a proceeding would lead to probable difficulties and to certain delay, which it was my object to avoid. Accordingly, the signing of the memorandum and the articles of association became largely a formal matter, and the original Council of fifteen appointed by the signatories has already been increased by co-optation to twenty-three, and may be further enlarged up to thirty members. A list of the present Council is enclosed herewith, and I shall be glad also to furnish on application a copy of the memorandum and articles of association, which it should be remembered have, in accordance with the usual practice in these cases, been drawn so as to give the widest possible powers, and not as a precise indication of the present or future policy of the College.

### A Voluntary Scheme of Representatives.

As I stated in my original letter, we have for the time being at least to rely upon a voluntary scheme of co-operation amongst the nurse-training schools throughout the kingdom; but, concurrently with the activities of the College as regards the training and certification of nurses and other women workers in hospitals, opportunities will be taken for friendly conference on the one hand with those who are altogether averse to State interference, and on the other with those societies whose primary object is the registration of nurses by Act of Parliament. To meet representatives of such societies a Registration Committee has already been appointed; and, whilst it would be idle to ignore the many difficulties which have to be overcome before the nursing profession wins the legal recognition it deserves, no one will rejoice more heartily than myself when, as the outcome of our common efforts, such a degree of substantial unanimity has been reached within the ranks as to enable us to present to Parliament an agreed Bill to gain legal status for the results attained by the combined activities of all who, working at present on different lines, have as their one objective the betterment of the nursing profession. In these aims I feel assured that we can rely upon the sympathy and all-important help of the medical profession, with which nursing is so closely allied.

### The College to be Self-governing and Democratic.

Another point upon which I have laid emphasis is that the College is ultimately to be self-governing. Primarily, it is true, the scheme is based upon the co-operation of the matrons and lady superintendents of the leading nursing schools, whose knowledge and experience in matters of curriculum and examination, supported and amplified by the work of the Consultative and Examination Boards, are made use of to initiate the undertaking. Accordingly the original Council has been thus nominated and co-opted, but in 1918, and every year afterwards, one-third of the members retire from office, and vacancies are filled by the votes of the members of the College, *i.e.*, from the general body of nurses upon the Register. To ensure for them an effective influence in the result there is provision for a postal vote, not dependent upon personal attendance at the annual ordinary meeting at which elections to the Council are made. Whilst, therefore, the College in its early years will be largely guided by the experience of the heads of the various training schools who form a majority of the first Council, it will, when it attains maturity, become broad-based and democratic in constitution.

### Uniformity of Curriculum and One Portal.

The third and fourth points upon which I have insisted are uniformity of curriculum and the one-portal system of examination, and it is just in these matters that the Council feel the urgent necessity of help and counsel from nursing schools, from the medical profession, from nursing associations, and from nurses in the active practice of their calling.

### The Solidarity of the Nursing Profession.

The solidarity of the nursing profession being a matter of paramount importance, it is essential that there should be only one certificate of general training for all nurses, wherever trained. At the present time, however, the nurses of the best London and provincial hospitals and infirmaries are able to make a living on the reputation of the nursing certificates they hold from their own schools, and thus have comparatively little inducement to enter for, and, incidentally, to pay fees for, any further qualifying examination. Hence the importance to the scheme of the countenance and active participation of the authorities of the leading nurse-training schools, and hence the provision, under stringent safeguards as to standard, for accepting the internal examinations of recognised schools as qualifying for the certificates of proficiency to be granted by the College. Moreover, such a concession avoids excessive centralisation, whilst it gives an incentive to backward institutions to improve their curricula, teaching facilities, and tests, so that they may gain the status of recognised schools, and thus be saved the necessity of sending their nurses elsewhere to sit for the College examinations.

### The Whole United Kingdom Included.

In view of the inclusion of *the whole* of the *United Kingdom* in the scheme, and the geographical difficulties thus introduced, power is taken in the articles of association to establish Local Boards, which may exercise in their respective areas "any of the powers, authorities, and discretions for the time being vested in the Council," and some progress has already been made in regard to Scotland.

### The Consultative Board—Its Constitution and Functions.

Reverting to the constitution and functions of the Consultative Board, for which, as I have already said, the Council now invite your managers to nominate not more than two persons, I would remark that under the articles of association it is to consist of "such number of persons as the Council shall from time to time determine," and to be "elected from amongst physicians, surgeons, hospital matrons and principal officers, superintendents of nursing, trained nurses, and persons interested in the relations between nurses and the public." Amongst the functions assigned to the Consultative Board are:—

(1) To deliberate in regard to any question submitted to it by the Council, and to report to the Council the result of the consultation thereon.

(2) On the initiative of any member of the Consultative Board to consider, if thought advisable, any subject within the scope of the memorandum of association; and, if thought advisable, to report thereon to the Council.

In order to ensure for the Consultative Board the fullest possible influence and authority in the College compatible with the position of the Council as the governing body, it is laid down that the Council shall always invite and receive a report from the Consultative Board before coming to a determination upon any of the following matters, viz.:—

(1) The courses of study and technical training for persons intended for the nursing profession.

(2) The conditions under which recognition may be extended to nursing schools.

### The Consultative Committee.

For facilitating the work of the Consultative Board the Council has formed a Consultative Committee, with power to add to its numbers from amongst persons appointed on the Board, so that business may be put before the full Board in such a way as not to make too great claims upon the time of its members, many of whom can only give occasional attendance in London.

### The Work of the Consultative Board.

From what has been said it will be evident that the work of the Board is chiefly of a professional character, but managers of hospitals may find that the revision of nursing curricula entails extra expense in the matter of lectures, class-rooms, and proper facilities for study. Consequently, some representation of the lay element in the management of hospitals and infirmaries appears to me to be desirable, in order that this point of view may receive due attention at the deliberations of the Board.

### Numbers and Representation on Board.

Until it is seen what response is made to this invitation, the Council is not in a position to elect the Consultative Board, or to fix its number, but, if the nominations are too numerous for efficient working, it may be necessary either to make a selection amongst them or to formulate some plan for representation on the Board by groups or districts. This, however, will be a matter for subsequent consideration, and meanwhile it is most important that as little time as possible should elapse before attacking some at least of the many problems which await solution before the College can take its place as the central organisation of the nursing profession throughout the United Kingdom. An early reply to this letter is requested.

